# Vascular phenotypes in early hypertension

**DOI:** 10.1038/s41371-022-00794-7

**Published:** 2022-12-17

**Authors:** Eleanor C. Murray, Christian Delles, Patryk Orzechowski, Pawel Renc, Arkadiusz Sitek, Joost Wagenaar, Tomasz J. Guzik

**Affiliations:** 1 School of Cardiovascular and Metabolic Health, University of Glasgow, Glasgow, UK; 2 Department of Biostatistics, Epidemiology and Informatics, University of Pennsylvania, Philadelphia, PA USA; 3 Department of Automatics and Robotics, AGH University of Science and Technology, Kraków, Poland; 4 Sano Centre for Computational Science, Krakow, Poland; 5 Department of Computer Science, AGH University of Science and Technology, Krakow, Poland; 6 Massachusetts General Hospital, Harvard Medical School, Harvard University Boston, Boston, MA USA; 7 Centre for Cardiovascular Science, University of Edinburgh, Edinburgh, UK; 8 Department of Medicine and Omicron Functional Genomics Laboratory, Jagiellonian University Collegium Medicum, Krakow, Poland

**Keywords:** Hypertension, Risk factors

## Abstract

The study characterises vascular phenotypes of hypertensive patients utilising machine learning approaches. Newly diagnosed and treatment-naïve primary hypertensive patients without co-morbidities (aged 18–55, *n* = 73), and matched normotensive controls (*n* = 79) were recruited (NCT04015635). Blood pressure (BP) and BP variability were determined using 24 h ambulatory monitoring. Vascular phenotyping included SphygmoCor® measurement of pulse wave velocity (PWV), pulse wave analysis-derived augmentation index (PWA-AIx), and central BP; EndoPAT™-2000® provided reactive hyperaemia index (LnRHI) and augmentation index adjusted to heart rate of 75bpm. Ultrasound was used to analyse flow mediated dilatation and carotid intima-media thickness (CIMT). In addition to standard statistical methods to compare normotensive and hypertensive groups, machine learning techniques including biclustering explored hypertensive phenotypic subgroups. We report that arterial stiffness (PWV, PWA-AIx, EndoPAT-2000-derived AI@75) and central pressures were greater in incident hypertension than normotension. Endothelial function, percent nocturnal dip, and CIMT did not differ between groups. The vascular phenotype of white-coat hypertension imitated sustained hypertension with elevated arterial stiffness and central pressure; masked hypertension demonstrating values similar to normotension. Machine learning revealed three distinct hypertension clusters, representing ‘arterially stiffened’, ‘vaso-protected’, and ‘non-dipper’ patients. Key clustering features were nocturnal- and central-BP, percent dipping, and arterial stiffness measures. We conclude that untreated patients with primary hypertension demonstrate early arterial stiffening rather than endothelial dysfunction or CIMT alterations. Phenotypic heterogeneity in nocturnal and central BP, percent dipping, and arterial stiffness observed early in the course of disease may have implications for risk stratification.

## Introduction

The demographic and lifestyle risk factors for hypertension are well recognised. Less well defined is associated cardiovascular dysfunction at the onset of the disease process, and early heterogeneity of cardiovascular phenotypes within incident hypertension. Routine clinical phenotyping of hypertension based primarily on BP endures, despite the availability of recognised surrogate markers of cardiovascular dysfunction such as endothelial and microvascular impairment, large artery stiffening, and arteriosclerosis [1]. Studies of vascular phenotyping have typically focused on functional and structural assessments related to complications of hypertension [2, 3]. Much less is known on the characteristics of these vascular phenotypes in early hypertension. Classically, endothelial dysfunction is considered as one of the earliest vascular disease traits in hypertension [4], that may precede arterial remodelling, vascular stiffness, micro-circulatory rarefaction and atherosclerosis [2]. These dysfunctional vascular traits are independent predictors of both hypertension and cardiovascular events, including myocardial infarction and stroke [2, 3, 5–7].

Despite their importance in advanced disease, it remains unclear if vascular traits such as endothelial dysfunction, vascular stiffness or carotid remodelling can be identified early, in treatment-naïve, incident hypertensive patients free of major comorbidities. Accordingly, we aimed to characterise vascular phenotypes of young, untreated patients with incident hypertension in comparison to normotension; and utilising machine learning approaches, to identify if patient heterogeneity is already present at this early stage of hypertension. Defining such clinical and vascular phenotypes may facilitate risk stratification and permit future precision medicine approaches in hypertension management.

## Methods

### Patient cohort

Inflammatension project (NCT04015635) was designed as a cross-sectional clinical and biomarker study of consenting patients from the West of Scotland aged 18 to 55 years with primary hypertension, and intention to match control participants on age, sex, and BMI. The study was approved by the West of Scotland Ethics committee. Sample size was based on a power calculation to detect differences in the immune system (NCT04015635), with consideration of published data detecting differences in vascular traits [8, 9]. Hypertensive participants were recruited based on office blood pressure (BP) greater than 140/90 mmHg. Researchers were blind to hypertensive status during the study visit, as final categorisation of participants to normotensive or hypertensive groups followed the study visit, determined by 24 h ambulatory blood pressure monitoring (ABPM) results, and defined in accordance with ESC/ESH definitions [10]: 24 h mean above 130/80 mmHg, or a daytime mean above 135/85 mmHg. Patient flow chart is available as Supplementary Fig. S1. Exclusion criteria included acute or chronic infections and inflammatory disorders; anti-hypertensive medications, known secondary hypertension, and BMI above 35, detailed criteria in Supplementary File 1. All study visits had start times between 8.30 and 10 am. Exercise that morning was avoided; patients attended a single clinical research facility (Glasgow, UK) fasted and avoided caffeine and cigarettes for a minimum 6 h. All studies were conducted in a quiet, temperature-controlled room and followed a pre-specified order as follows in the Clinical Measurements section, other than ABPM, which commenced at the close of the study visit.

## Clinical measurements

### Blood pressure

Office BP was measured in the sitting position after 5 min of rest, by a validated sphygmomanometer (OMRON Digital Automatic Blood Pressure Monitor HEM-907, Kyoto, Japan), with an appropriately sized cuff. Three measurements taken were summarised in data reporting as a mean value. ABPM performed for 24 h comprised twice-hourly BP measurements throughout the day in accordance with most recent European practice guidelines [11], and hourly overnight (22.00 until 7.00, Spacelabs’ 90217 A, Spacelabs Healthcare, Hertford, UK). A failed recording resulted in one repeat attempt. Data are presented as average 24 h values, average daytime values, and average night-time values for systolic and diastolic BP. Hypertension was defined as 24 h mean above 130/80 mmHg, or a daytime mean above 135/85 mmHg [10]. Nocturnal systolic dipping was defined as a nocturnal decrease in the average ambulatory SBP by 10% or more, diastolic dipping similarly defined [10]. BP variability was defined as systolic and diastolic BP standard deviation in ABPM. Heart rate variability (heart rate range determined by maximum and minimum recorded heart rate) was also recorded.

### Peripheral artery tonometry (PAT)

Measured using EndoPAT™-2000 (Itamar Medical, Israel). A 5-min baseline measurement was followed by 5-min proximal arterial occlusion through supra-systolic inflation of a blood pressure cuff. Occlusion was released and 5 min of post-ischaemic vascular responsiveness were recorded. The ratio of the signal pre- and post-occlusion generated a reactive hyperaemic index (RHI), automatically natural log transformed (LnRHI). Pulse waveforms obtained during baseline measurement provided mean heart rate and Augmentation Index corrected to a heart rate of 75 beats/min (AI@75). Contra-lateral arm was used as control.

### Flow mediated dilatation (FMD)

UNEX EF38G model (Unex, Nagoya, Japan) permitted ultrasound visualisation of the brachial artery and automated measurement of arterial diameter. The cuff was placed on the contra-lateral arm to PAT to prevent repeat-occlusion endothelial ‘priming’, with a 15-min time interval between techniques. Baseline arterial diameters were recorded; the cuff was then inflated to 50 mmHg above systolic pressure (max 250 mmHg) distal to the ultrasound for 5 min, and arterial diameter was reassessed following release of cuff. Arterial diameter variation across the cardiac cycle was corrected for using integrated heart rate recording. Integrated software analysis provided percent FMD, rest, baseline, and maximum arterial diameter measurements with wall-tracking capability.

### Pulse wave analysis (PWA)

Performed supine after 10 min of rest with SphygmoCor XCEL (Atcor medical, West Ryde, Australia). An appropriately sized cuff was placed on the upper arm, acquiring two brachial BP measurements with a 1-min interval. The cuff then partially inflated to capture the brachial artery waveform for 10 s, with automated analysis and integrated quality control. The report included central systolic, central diastolic, and Augmentation index (AIx). Two measurements were performed with a mean value used in data analysis.

### Pulse wave velocity (PWV)

Determined using SphygmoCor XCEL. With the participant supine and rested, a BP cuff was applied to the femoral artery. The subtraction method was used to estimate the length of the aorta. Once the pressure probe detected the common carotid artery pulse-wave sufficiently, the leg cuff automatically inflated. Both pressure waves were captured simultaneously based on the systolic upstroke. Automated PWV calculation was reported in metres per second (m/s), with quality control analysis indicating pulse-to-pulse variability. If a repeat measure differed by >1 m/s, then a third measure was performed and the two with greatest concordance were recorded.

### Carotid intima media thickness (CIMT)

B-mode ultrasonography of the right carotid artery performed with Acuson Sequoia c512 (Siemens, Germany). Following published guidance, [12] the patient was positioned supine and the common carotid artery wall was visualised at both 90 and 135 degrees with the carotid bifurcation included for reference, and images saved in reference to the R wave of the ECG, corresponding to end-diastole. CIMT was measured on high-resolution 1.2 × 1.2 cm image, as the distance between lumen–intima interface and the media–adventitia border. Analysis was performed off-line in batches, by a single assessor, utilising Carotid Studio (QUIPU, Version 3.6.0, 2019, Italy) software with multiple data points along the wall of the vessel captured and an average measurement generated. Due to access limitations related to COVID-19, this assessment was possible only in 93 patients.

### Physical activity questionnaire

Self-reported physical activity was assessed using the validated open-access ‘International Physical Activity Questionnaire (IPAQ; short version)’ [13]. The final score was expressed as MET-min per week (MET level x minutes of activity x events per week).

### Cardiovascular risk score

The InterHeart Risk Score Questionnaire is a self-reported scoring system to predict cardiovascular events and validated in diverse populations [14]. The output is a score from 0 to 48.

### Vascular data software analysis

Both CIMT and FMD analysis were performed off-line in batches, by a single assessor, blinded to the participant groups.

## Data analysis and machine learning

Analysis was performed on Minitab version 19 and Microsoft Excel (2013). Statistical significance was assumed for *P* < 0.05. Between-group comparisons employed 2-tailed *T*-test, Mann–Whitney or Moods’ test depending on distribution of the data. Analysis of continuous variables was based on correlation and multi-variable regression modelling (Table S2 and Supplementary File 2) to assess for associations between BP and vascular measures, and if associations remained significant after adjustment.

For machine learning approaches applied to the hypertensive group, variables that were highly correlated or with a substantial number of values missing were removed, giving 19 features in total. Missing data points were imputed from within-sex group median values, and data scaled to a columnar mean of 0 and standard deviation of 1 (*z*-score). Distinct phenotypes were identified using Uniform Manifold Approximation and Projection (UMAP) as a dimensionality reduction technique, combined with Spectral Biclustering. Taking each in turn, UMAP learns the manifold structure of data by graphing neighbouring samples, where edge weights reflect similarities (or distances) between nodes according to a given metric (e.g., Euclidean distance). The embedded data is then projected to lower-dimensional space while preserving its topological structure. The optimal number of clusters to capture distinct groups was defined by Silhouette score. Shapley (SHAP) values were computed to better interpret discriminating features for each of the UMAP clusters.

To better capture homogenous patterns appearing in the data, we utilised spectral biclustering. This technique assumes the data has a checkerboard structure, with the number of partitions in both dimensions as input. Each row is thus assigned to the same number of biclusters as the number of column partitions, and vice-versa. While classical clustering focuses on detecting ‘global’ similarities based on all features, biclustering reveals patterns containing ‘local’ subsets of features and subgroups of patients to generate the subgroups. These were subsequently analysed for between group differences with use of box-plots and ANOVA. Detailed analytical methodology is included in Supplementary File 3.

## Results

### Clinical characteristics

Demographic and cardiovascular characteristics are presented in Table 1, demonstrating young age (median 39 ± 14 years) and closely matched groups, only differing on BMI (Table 1). Some measures of cardiovascular variability such as SBP SD differed by hypertension status, though others (nocturnal BP dipping and HR range) did not. Arterial stiffness was greater in the hypertensive group (Table 1), consistent across the independent techniques (PWV, PWA-AIx, and EndoPAT-2000 derived AI@75), and remained statistically different following adjustment for age, sex, and BMI. Similar was true for arterial Systolic- and Diastolic-Central Pressures (SCP and DCP). In contrast, neither measures of endothelial function (LnRHI and % FMD) nor CIMT differed significantly between normotensive and incident hypertensive groups.

**Table 1:** Clinical characteristics of participants by blood pressure group.

	Normotension = 79	Hypertension = 73	*P* value
Male sex (%)	41 (52)	41 (56)	0.598
BMI, kg/m2, mean (SD)	26.3 (4.8)	28.8 (4.1)	0.001
Age, years, median (IQR)	39 (12)	39 (14)	0.83
SBP, mmHg, Mean (SD)	124.6 (13.9)	146.8 (15.3)	<0.0001
DBP, mmHg, mean (SD)	80.0 (10.5)	93.0 (11.8)	<0.0001
BAME Ethnicity *N* (%)	8 (11.0)	11 (15.1)	0.50
Smoking *N* (%)	6 (8.2)	1 (1.4)	n/a
SBP24, mmHg, mean (SD)	115.7 (8.9)	141.1 (9.0)	<0.0001
DBP24, mmHg, mean (SD)	72.7 (7.0)	88.1 (7.9)	<0.0001
SBP SD, mmHg, median (IQR)	9.6 (2.7)	11.4 (3.0)	<0.0001
DBP SD, mmHg, median (IQR)	8.0 (4.1)	9.0 (3.6)	0.07
SBP day, mmHg, mean (SD)	119.7 (9.5)	145.4 (8.7)	<0.0001
DBP day, mmHg, mean (SD)	76.4 (7.4)	91.5 (7.7)	<0.0001
SBP night, mmHg, mean (SD)	102.9 (10.5)	124.7 (13.3)	<0.0001
DBP night, mmHg, mean (SD)	61.9 (7.9)	74.2 (11.1)	<0.0001
% dip SBP mean (SD)	11.7 (5.3)	12.0 (6.3)	0.75
% dip DBP mean (SD	15.5 (6.2)	12.0 (6.3)	0.66
Nocturnal dip (%)	43 (58.9)	44 (60.3)	0.57
HR range day, median (IQR)	40 (26.5)	34 (13.3)	0.18
HR range night, median (IQR)	22 (15)	19 (11.5)	0.79
CIMT mm mean (SD)	0.55 (0.12)	0.54 (0.12)	0.58
FMD % mean (SD)	4.9 (4.25)	6.1 (4.05)	0.40
Rest diameter mean (SD)	3.94 (0.74)	4.2 (0.77)	0.07
Max diameter mean (SD)	4.26 (0.83)	4.5 (0.76)	0.04
LnRHI mean (SD)	0.69 (0.28)	0.78 (0.31)	0.06
AIx median (IQR)	6 (18)	14 (31)	0.06
HR mean (SD)	63.1 (10.2)	67.1 (10.6)	0.02
AI@75% median (IQR)	−1.00 (24.2)	9.00 (28.5)	0.01
PWV m/sec mean (SD)	6.57 (1.29)	7.50 (1.7)	<0.0001
PWA AIx mean (SD)	9 (28.0)	11.75 (27.3)	0.05
SCP mmHg mean (SD)	113.1 (12.5)	133.0 (14.0)	<0.0001
DCP mmHg mean (SD)	77.9 (9.5)	93.0 (10.6)	<0.0001
IDQ median (IQR)	7.0 (8.0)	12.0 (6)	<0.0001
IPAQ median (IQR)	2706 (2629)	1896 (1402)	0.01

*DBP* diastolic blood pressure, *BAME* Black Asian and Minority Ethnic, *SD* standard deviation, *HR* heart rate, *CIMT* carotid IMT, *LnRHI* log of reactive hyperaemia index, *AI@75%* EndoPAT2000 derived augmentation index adjusted for heart rate, *PWV* pulse wave velocity, *PWA* pulse wave analysis, *PWA AIx* augmentation index derived from PWA, *SCP* systolic central pressure, *DCP* diastolic central pressure, *IDQ* interheart diet score, *IPAQ* International Physical Activity Questionnaire.

*Indicates statistical significance following adjustment for age, sex and BMI in regression analysis with BP as a categorical response. Hypertension group based on 24 h systolic blood pressure (SBP) above 130 mmHg or daytime SBP above 135 mmHg; Normotension group had BP values below these thresholds.

### Associations between cardiovascular parameters and BP

**Fig. 1:**
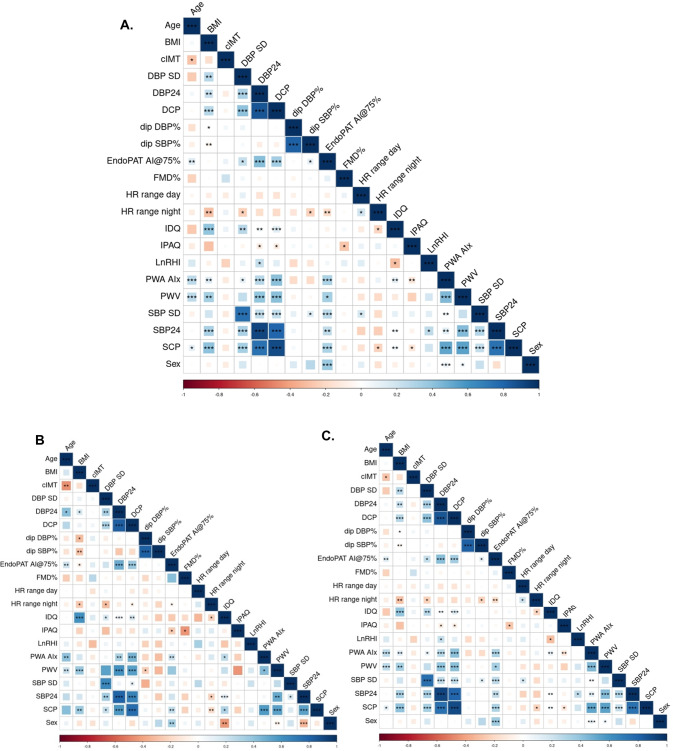
Correlation matrix of Cardiovascular and blood pressure parameters. All studied participants (Panel **A**), and separately by normotension and hypertension groups (Panels **B** and **C**). Colour and colour intensity indicate r values i.e., direction and strength of correlation (red negative correlation, blue positive), as per *X* axis. *P < 0.05 **P < 0.01 ***P < 0.001. BMI body mass index; CIMT carotid intima media thickness, DBP diastolic blood pressure, SD standard deviation, DCP diastolic central pressure, AI@75% AIx adjusted for heart rate, FMD% percent flow-mediated dilatation, HR heart rate, IDQ interheart diet score, IPAQ International Physical Activity Questionnaire, LnRHI logarithmic transformation of reactive hyperaemia index, PWV pulse wave velocity, PWA pulse wave analysis, AIx augmentation index, SBP systolic blood pressure, SCP systolic central pressure.

Correlations were analysed within the whole study population (Fig. 1A; Table S1), and separately in normotensive and hypertensive groups (Fig. 1B). FMD did not correlate with BP variables. LnRHI demonstrated positive BP association restricted to hypertensive patients (24 h SBP *r* = 0.41, 24 h DBP 0.23, both *P* < 0.05), Fig. 1B, C. Correlation of arterial stiffness measures with BP was consistent across various techniques (PWV, AI@75, PWA-AIx), and across different BP parameters, most pronounced was PWV and 24 h SBP (*r* = 0.43, *P* < 0.001, Table S1). Figure 1B, C illustrates that correlations were similar between hypertension and normotension for PWV and PWA-AIx, but diverged for AI@75, with 24 h SBP reaching significance only in the hypertensive population. The correlation matrix (Fig. 1) highlights that SCP and DCP demonstrate broad association with both arterial stiffness and 24 h BP parameters, preserved across normotensive and hypertensive groups and all statistically significant. CIMT did not demonstrate correlation with BP variables.

### Concordance of vascular parameters in assessing functional traits

Techniques measuring arterial stiffness demonstrated collinearity, with strongest correlation between AI@75 derived from EndoPAT-2000, and AIx as measured by SphygmoCor (*r* = 0.50, *P* < 0.001), PWV demonstrated weaker association with both (Table S1 and Fig. 1). Regarding measures of endothelial function, % FMD did not correlate with LnRHI across the whole cohort (*r* = 0.05, *P* = 0.56). Though participants with ‘abnormal’ LnRHI results (<0.51) did demonstrate higher % FMD (7.5% vs 5.8%, *P* = 0.03). Neither LnRHI nor % FMD correlated with measures of arterial stiffness or CIMT (Table S1 and Fig. 1).

### Masked and white coat hypertension

Thirteen of 152 participants (9%) had elevated office BP but ABPM in the normal range, so called ‘white coat’ hypertension (WCH) [15]. 18/152 (12%) had 24 h average SBP exceeding 130 mmHg, but normal range office SBP, so called ‘masked hypertension’ (MHN). These rates are similar to those reported by others [16]. Table S3 illustrates that age and sex were distributed evenly across NTN, HTN, WCH, and MHN subgroups; other than a male dominance in MHN (15 of 18, 83%, *P* = 0.04), and BMI was higher in the sustained HTN group, *P* = 0.004. Between group differences were apparent both for measures of arterial stiffness (PWV, PWA-AIx, EndoPAT-2000-derived AI@75), and central pressures, all *P* ≤ 0.001. WCH subgroup demonstrated the greatest arterial stiffening, concordant across the different techniques; in contrast, MHN values were in the normotensive range, Table S3. Systolic and DCP for both WCH and MHN (123/79 and 136/83 mmHg, respectively), demonstrated values intermediate to NTN and sustained HTN groups (114/71 and 144/90 mmHg respectively), *P* < 0.001, Fig. S2; the association of central and brachial pressures across these subgroups is included in Supplementary Fig. S2. Measures of endothelial function, CIMT and percent nocturnal BP dip did not differ between groups i.e., WCH and MHN are not simply subgroups determined by nocturnal dipping status.

### Heterogeneous sub-phenotypes of early hypertension

**Fig. 2:**
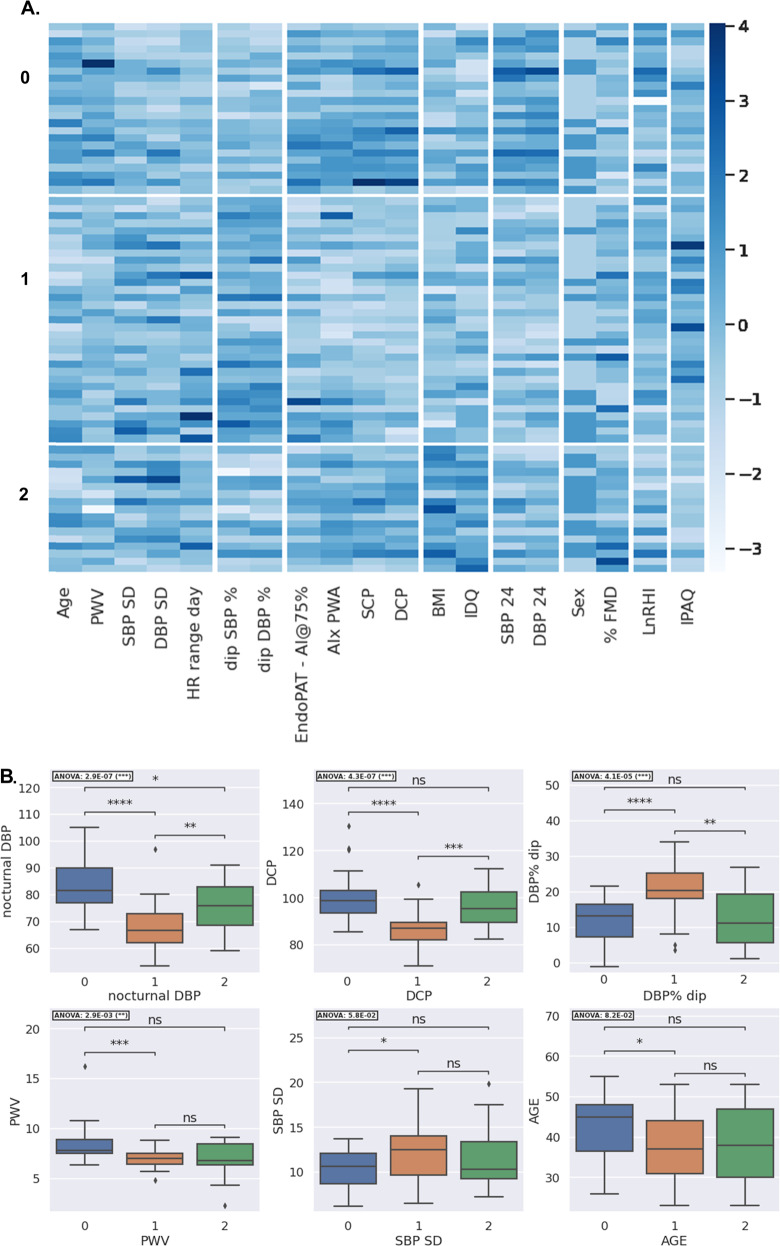
Cluster analyses of the hypertensive group. Spectral Biclustering with three subgroups of patients and eight groups of features (Panel **A**). Boxplots of key features discriminating bicluster groups (Panel **B**). Both panels: Group 0 ‘arterially stiffened’ hypertension, Group 1 ‘vasoprotected’ hypertension, Group 2 ‘non-dippers’. SBP24, 24 h average systolic BP; DBP24, 24 h average diastolic BP; SBP SD standard deviation i.e., variability of systolic BP; DBP SD, standard deviation i.e., variability of diastolic BP; FMD flow mediated dilatation, BMI body-mass index, dip percentage reduction from day to night BP, LnRHI logarithmic reactive hyperaemia index, AIx EndoPAT-2000-derived augmentation index, AI@75% AIx adjusted for heart rate, HR range day daytime heart rate range, PWV pulse wave velocity, PWA pulse wave analysis, SCP systolic central pressure, DCP diastolic central pressure.

Within the hypertensive group, Spectral biclustering identified three distinct groups of patients (rows), characterised by eight groups of features (columns) (Fig. 2). FMD, LnRHI, CIMT, and HR range showed no differences between bicluster groups, consistent with analyses reported above. The remaining demographic and vascular features characterise distinct phenotypes of incident hypertension as follows.

Group 0 subjects (*n* = 23) could be considered as ‘arterially stiffened’ hypertension. These patients trended toward being older (NS), 57% male, with a lower mean physical activity score (data and ANOVA analyses are reported fully in Table 2). Average BP values were highest for this group, seen across 24 h BP (149 ± 9 / 95 ± 8 mmHg, ANOVA *P* < 0.0001), day and night mean values, office BP, and central BP (143 ± 15 systolic, 101 ± 11 diastolic, mmHg, both *P* < 0.0001). BP variation (SD) was lowest, % nocturnal dip was reduced (systolic dip 9.3 ± 4%), and a lower proportion of masked hypertension (MHN) patients were present (3 of 23, 13.0%). Arterial stiffening was apparent, with highest PWV (8.5 ± 2.1 m/s, *P* = 0.003) and AI@75 (17.4 ± 17, *P* = 0.039 (Table 2). FMD values were lowest (5.06 ± 3.15%), but did not reach statistical significance (*P* = 0.059).

**Table 2:** Comparison of hypertensive cluster groups: Group 0 ‘arterially stiffened’ hypertension, Group 1 ‘vasoprotected’ hypertension, Group 2 ‘non-dippers’.

Cluster group	FMD	LnRHI	CIMT	PWV	AI@75 EndoPAT	PWA-AIx	SCP	DCP	BMI	IPAQ	AGE	Female Sex	White Ethnicity
0 *N* = 23	5.06 (3.15)	0.83 (0.37)	0.56 (0.13)	8.50 (2.13)	17.38 (16.95)	17.41 (14.91)	142.59 (15.19)	100.68 (11.28)	27.75 (3.22)	2336.39 (1727.67)	42.61 (7.76)	10 (43%)	18 (78%)
1 *N* = 33	6.56 (3.88)	0.81 (0.24)	0.53 (0.11)	7.04 (0.86)	3.48 (22.03)	4.73 (18.93)	125.05 (8.00)	86.56 (6.59)	27.32 (3.01)	4150.06 (4087.56)	37.39 (8.71)	11 (33%)	32 (97%)
2 *N* = 17	8.03 (4.38)	0.67 (0.35)	0.51 (0.08)	7.01 (1.88)	6.32 (13.83)	19.59 (12.00)	136.06 (13.35)	95.56 (8.62)	33.06 (4.21)	1077.81 (819.15)	38.12 (9.96)	11 (65%)	12 (71%)
ANOVA	0.0591	0.2892	0.6646	0.0029	0.0391	0.0035	<0.0001	<0.0001	<0.0001	0.003	0.0823	0.1061	0.1159
**Cluster group**	**SBP24**	**DBP24**	**SBP day**	**DBP day**	**SBP noct**	**DBP noct**	**SBP % dip**	**DBP % dip**	**SBP SD**	**DBP SD**	**Mean HR**	**HR range day**	**HR range night**
0	147.67 (8.71)	94.39 (7.45)	150.65 (9.06)	96.57 (7.61)	134.49 (10.30)	83.20 (9.58)	9.29 (4.17)	11.90 (5.77)	10.24 (2.05)	8.32 (2.48)	68.10 (9.22)	32.35 (6.66)	21.57 (10.29)
1	137.25 (6.52)	84.67 (6.56)	142.90 (6.76)	89.22 (6.99)	117.15 (10.86)	67.69 (8.78)	15.45 (5.73)	20.75 (7.35)	12.19 (3.08)	9.34 (2.50)	62.48 (9.52)	41.56 (19.87)	23.00 (9.33)
2	139.65 (8.99)	86.24 (5.49)	143.18 (9.07)	89.12 (6.05)	127.44 (13.73)	75.41 (8.70)	8.95 (7.06)	12.66 (8.55)	11.53 (3.53)	10.51 (2.98)	74.73 (10.09)	36.88 (14.31)	23.27 (11.74)
ANOVA	<0.0001	<0.0001	0.0016	0.0004	<0.0001	<0.0001	0.0001	<0.0001	0.0585	0.038	0.0007	0.0987	0.8503

Mean value and standard deviation of cardiovascular parameters by cluster groups, with ANOVA assessment of between group difference and R^2^ adjusted values indicating strength of the model. SBP24, 24 h average systolic BP; DBP24, 24 h average diastolic BP; SBP SD, standard deviation i.e., variability of systolic BP; DBP SD, standard deviation i.e., variability of diastolic BP.

*BMI* body-mass index, *dip* percentage reduction from day to night BP, *LnRHI* logarithmic reactive hyperaemia index, *FMD* flow-mediated dilatation, *AI@75%* EndoPAT-2000-derived augmentation index adjusted for heart rate, *HR range day* daytime heart rate range, *PWV* pulse wave velocity, *PWA* pulse wave analysis, *PWA-AIx* pulse-wave analysis derived augmentation index, *SCP* systolic central pressure, *DCP* diastolic central pressure.

Group 1 included subjects (*n* = 33) with pronounced nocturnal dipping, so far ‘vaso-protected’ from major vascular impairment. These predominantly male (67%) patients appear fitter, with highest physical activity index, low resting HR, wider HR range, and SBP SD. BP values (Table 2) were lowest including 24-h BP 137 ± 9 / 85 ± 7 mmHg (both *P* < 0.0001), day/night averages, office BP, and central BP; greatest nocturnal dip was also seen (systolic 15.4% ±4.2, diastolic 20.7% ±5.8, both *P* ≤ 0.0001). MHN patients were over represented (13 of 33, 39.3%, NS). This group showed lowest measures of arterial stiffness by PWV or AIx (Table 2).

Group 2 included ‘non-dippers’ (*n* = 17), Table 2 demonstrating lowest physical activity, female predominance, and more diverse racial background. They demonstrated intermediate BP measures across parameters of 24 h BP (140 ± 9/86 ± 6 mmHg, both *P* < 0.0001), daytime BP, night-time BP, office BP, and central BP and had a low proportion of masked hypertension (MHN) patients (2 of 17, 11.7%, NS). Mean nocturnal dip values were low (9.0 ± 7.1% systolic, 12.6 ± 8.6% diastolic, both *P* ≤ 0.0001). Regarding arterial stiffness, AIx derived from PWA was elevated, but resting HR was also higher and the HR-adjusted AI@75 as well as PWV were intermediate. Group 3 FMD values were highest but did not reach statistical significance. DBP SD was highest and SBP SD intermediate, despite reduced nocturnal dipping. Among all these between-group differences, nocturnal and central BP were the statistically strongest parameters (all *P* < 0.0001, Table 2).

For further validation of the machine learning model, we analysed SHAP values computed from the dataset with UMAP dimensionality reduction techniques. Central BP was a key driver across the three clusters, augmentation index in clusters 0 and 1, 24 h DBP in clusters 0 and 1; SBP % dip and cardiovascular variation (SD, heart rate range) in clusters 0 and 2; PWV in cluster 1, and demographic features in cluster 2. Other features important for given clusters can be found in Figs. S3 and S4.

## Discussion

Characterising a unique cohort of newly diagnosed, untreated, hypertensive subjects without comorbidities or target organ damage; arterial stiffness appears to be the only vascular functional trait increased early in hypertension and clearly related to increased blood pressure. FMD-determined endothelial function was not altered, while EndoPAT™-2000® positively correlated to SBP, showing a possible compensatory response. Machine learning approaches identified a clear heterogeneity of newly diagnosed hypertensive subjects, with identification of three phenotypes based primarily on nocturnal- and central-BP, percent dipping, and arterial stiffness measures.

Longitudinal studies of both healthy and co-morbid populations evidence arterial stiffening in newly diagnosed hypertension. Diverse measurement techniques studied include carotid-femoral and brachial-ankle PWV, augmentation index, and carotid elasticity, as determinants of longitudinal BP increase and as independent predictors of incident hypertension [17–20]. The relationship strengthens in established or uncontrolled hypertension, where 90% display elevated PWV [5]. Risk of cardiovascular events also rises with increasing arterial stiffness; [3, 5] meta-analysis of 17 longitudinal studies suggesting that this risk is amplified by other evidence of hypertension-mediated organ damage (HMOD) [21]. However, the sequential nature of arterial stiffening and hypertension remains debated [22, 23]. This debate may arise from data sampling periods, with the relationship between arterial stiffness and BP evolving over time. For example, UK BioBank data suggests midlife DBP as the strongest predictor of arterial stiffness progression, transitioning to increased stiffness and a falling DBP [24]. Capturing an incident hypertensive population such as we describe is therefore critical when exploring early interactions of BP and vascular function.

In contrast, measures of endothelial function were interesting in their absence of collinearity, or association with hypertension or arterial stiffness in whole-cohort analyses. FMD and EndoPAT™-2000® are not inter-changeable techniques [25], reflecting macro- and micro-vascular function, respectively. Longitudinal data have demonstrated increased prevalence of hypertension and elevated PWV in impaired FMD [26, 27], but contrasting evidence from both healthy and untreated hypertensive individuals shows no association with incident hypertension [27] or arterial stiffness, and suggests that no excess of microvascular dysfunction exists among otherwise healthy incident hypertensive individuals [28–30]. Indeed, the hypertensive subgroup demonstrated positive correlation of LnRHI with 24 h SBP, suggesting that the endothelium may demonstrate early compensation in hypertension and arterial stiffening. No association existed between CIMT and BP, despite well-established evidence supporting the relationship [7, 31], suggesting that this incident population have not yet developed clinically detectable atherosclerosis. This is supported by other data, even stroke patients failing to demonstrate elevated CIMT if younger in age [20, 32].

Despite central BP correlating with brachial measures across the whole group (Fig. 1 and Fig. S2), our subgroup of patients with WCH demonstrated central BP and arterial stiffness estimates in excess of those with sustained hypertension, and MHN vascular characteristics were akin to normotension. These findings are consistent with published research; firstly, others have also observed features of vascular damage in WCH [33, 34] that may relate to increased sympathetic drive, in turn linked to subclinical alterations of organ structure and function [35]. Conversely, MHN has been associated with only marginally elevated markers of target organ damage in comparison to normotension, that may be more pronounced in out-of-office settings [33, 36]. In published literature, equipoise remains regarding the predictive value of central BP above brachial [37, 38], superior predictive value of central BP in CIMT and future cardiovascular outcome reported by some [39–41]. Alternatively, data may reflect a limitation of office measurements in failing to accurately reflect ‘ambulatory’ arterial stiffness and central pressure, similar to the limitations of office BP [33]. External validation should include ambulatory PWA to assess this possibility. Definitions based on office and ambulatory BP also carry a risk of confounding from sleep disturbance causing loss of nocturnal dipping [42]. However, no such relationship was seen within our data, indeed MHN was *under*-represented in the ‘non-dipper’ bicluster.

Robustness of the data is limited by the restricted sample size, and by the higher BMI in the hypertensive group, which is known to influence cardiovascular function, hence inclusion of BMI in adjusted analyses. Although researchers were blind to ABPM result and final grouping (ABPM being undertaken at the end of the study visit), bias may have arisen as the office BP was recorded during the visit. This was however unavoidable, as it forms an integral component of the vascular studies. Furthermore, it is challenging to ascertain if associations between arterial indices are true relationships, or rather demonstrate co-dependency on BP. Of note, earlier ABPM guidelines advised additional ABPM readings per hour compared to current accepted practice [43]; this could potentially have increased accuracy of BP variability measurements. External validation would partially counter these limitations; however, whilst other studies report hypertensive phenotypes, they have been limited by determination of defining characteristics a priori [44]. We believe strengths of this study are avoidance of pharmaceutical and co-morbidity confounders, and the application of advanced machine learning techniques.

UMAP and bicluster machine-learning methodologies demonstrated concordant results regarding key features of central and nocturnal BP values, nocturnal dipping pattern, and measures of arterial stiffness; supporting the validity of the data. The detected biclusters were also broadly consistent with results observed in basic statistical analysis, and carry translational clinical implications as follows. Group 0 ‘arterially stiffened’ hypertension, were also sedentary, with greatest elevation in BP parameters, and both reduced nocturnal dip and BP variability. These features may suggest a longer duration of undiagnosed hypertension. A lower threshold commencing primary prevention (lifestyle and pharmacological) may benefit this group. Group 1, ‘vaso-protected’ hypertension, were fitter, with lowest BP measures, preserved nocturnal dip, least evidence of arterial stiffness. The group could represent hypertension earlier in its’ natural course, ABPM key for diagnosis with 39% having MHN. Higher SBP variability and daytime HR range may reflect more physical activity or alternatively a strong sympathetic drive, be it social stresses, or stimulants such as caffeine, which should be identified and targeted as part of management. Group 2, ‘non-dippers’, also demonstrated elevated BP variability despite loss of nocturnal dip, but MHN was rare. Genotypic differences may be present, as group 2 were female dominated in comparison to the other groups and ethnically more diverse. Non-dipping status has been linked to risk of CVD and arterial stiffness in studies of older participants [45, 46]. As such, lifestyle interventions and bedtime dosing of anti-hypertensives may reduce future cardiovascular risk. In the clinical setting, obtaining this level of additional testing is not routinely possible. We propose that investing in such phenotyping at the point of diagnosis will offer clinical value, if translation into personalised approaches (as outlined above) improves BP control, risk stratifies patients, and delays HMOD and cardiovascular disease.

## Conclusions

Hypertensive disease progression involves early arterial stiffness, already detectable in this newly diagnosed, young, primary hypertensive group in comparison to normotensive controls. Carotid atherosclerosis and impairment in endothelial function were not detected. WCH patients demonstrated arterial stiffening in excess of sustained hypertension; MHN vascular characteristics were akin to normotension. We further conclude that unsupervised machine learning is a valuable analysis tool, offering deeper clinical insights into nuances between hypertensive phenotypes, here driven by nocturnal and central BP, percent dipping, and arterial stiffness. Given the prognostic value of these parameters, such phenotypes may have important clinical implications for disease progression and individualised care.

## Summary

### What is known about this topic

- The clinical impacts of hypertension are varied between individuals, but vascular dysfunction is common in advanced hypertension.
- Widely accepted phenotypic patterns include white-coat, masked, and non-dipper hypertension.

### What this study adds

- Arterial stiffening is already apparent in incident patients with primary hypertension.
- Nocturnal and central BP, and arterial stiffness are key parameters differentiating heterogeneity of hypertensive phenotypes.
- Machine learning techniques can maintain granularity of the data, offering deeper clinical insights.

## Supplementary information

SUPPLEMENTARY MATERIAL CLEAN

## Data Availability

Supplementary files contain additional data and figures. All raw data generated and analysed during the current study are available from the corresponding author on reasonable request.
