# Supplementary material for: Vascular phenotypes in early hypertension
Source: J Hum Hypertens. 2022 Dec 17;37(10):898–906. doi: 10.1038/s41371-022-00794-7 (PMC9758678; doi:10.1038/s41371-022-00794-7)
Supplement: Supplementary file 1 — SUPPLEMENTARY MATERIAL CLEAN [file 41371_2022_794_MOESM1_ESM.docx]

**SUPPLEMENTARY MATERIAL**

**Supplementary File 1. Inflammatension inclusion and exclusion criteria.**

**Inclusion criteria:**

- Age between 18-55 years
- Cases: Office blood pressure ≥140 and ≥90.

Controls: Office blood pressure <140 and <90; age, sex and BMI matching to cases.

**Exclusion criteria:**

(a) Age >55 years old;

(b) Secondary hypertension (including e.g. adrenal tumours, phaeochromocytoma, renal artery stenosis; thyroid disease)

(c) Acute inflammatory disorders incl. flu, rhinitis, sinusitis etc. within 3 weeks; hospitalization with an inflammatory condition within the past 3 months; Life expectancy of < 3 years; History of alcohol/substance abuse

(d) Allergic disorders; chronic infections, COPD, tuberculosis; hepatitis B or C; pneumonitis, bronchiectasis; pericardial or pleural effusion, ascites; liver disease;

(e) Chronic inflammatory/autoimmune conditions such (e.g. SLE, rheumatoid arthritis, ulcerative colitis/Crohn's disease; non-basal cell malignancy or myelo- or lymphoproliferative disease within the past 5 years; known HIV+; Immunizations (3 months); pulmonary hypertension;

(f) Pregnancy, nursing;

(g) History of symptomatic coronary artery disease (events) or heart failure;

(h) BMI >35, diabetes/glucose intolerance (fasting glucose, HbA1; testing, glucose challenge where indicated);

(i) Known albuminuria/microalbuminuria; GFR<60mL/min/1.73m2*.*

(j) Any chronic concurrent treatment. Use of systemic or local steroids / immunosuppressive agents (within 6 months) of the inclusion; current (within past 3 months) use of anti-hypertensive medication;

(k) Major depressive illness or other psychiatric conditions.

(l) Participants who decline participation in the study or who are unable to provide informed consent

**Figure S1. Inflammatension patient cohort, illustrated in a flow chart.**

BP as recruited:

NTN N=65

HTN N=89

Excluded N=2

NTN N=79

HTN N=73

Groups determined by ABPM:

Sus HTN N=55

WCH N=13

NTN N=66

MHN N=18

Phenotypic groups:

2 N=17 Non-dippers

1 N=33 Vaso-protected

0 N=23 Arterially stiffened

Cluster groups:

BP, blood pressure; ABPM, ambulatory BP monitoring; NTN, normotension; HTN, hypertension; WCH, white-coat hypertension; MHN, masked hypertension; SusHTN, sustained hypertension.

**Table S1. Pearson correlation (r values) of measures of vascular function with blood pressure (BP) variables, all participants.**

|  | LnRHI | AI@75 | % FMD | PWV | PWA-AIx | SCP | DCP | CIMT |
| --- | --- | --- | --- | --- | --- | --- | --- | --- |
| SBP24 | 0.22^#^ | 0.34* | 0.035 | 0.40* | 0.26^#^ | 0.73* | 0.75* | 0.008 |
| DBP24 | 0.17^#^ | 0.38* | 0.029 | 0.46* | 0.37* | 0.74* | 0.84* | 0.044 |
| MAP | 0.20^#^ | 0.35* | 0.032 | 0.45* | 0.34* | 0.76* | 0.82* | 0.029 |
| SBP day | 0.24^#^ | 0.29^#^ | 0.025 | 0.38* | 0.26^#^ | 0.73* | 0.73* | 0.004 |
| DBP day | 0.21^#^ | 0.38* | 0.022 | 0.43* | 0.35* | 0.73* | 0.82* | 0.051 |
| SBP noct | 0.17^#^ | 0.15^#^ | 0.045 | 0.32* | 0.20^#^ | 0.60* | 0.65* | 0.035 |
| DBP noct | 0.10 | 0.30^#^ | 0.021 | 0.42* | 0.33* | 0.59* | 0.72* | 0.06 |
| dip SBP % | -0.005 | 0.18 | -0.037 | 0.038 | 0.03 | 0.049 | -0.03 | -0.043 |
| dip DBP % | 0.051 | 0.04 | 0.017 | -0.09 | -0.05 | -0.002 | -0.08 | -0.029 |

* indicates P<0.001, ^#^ indicates P<0.05. SBP24, 24 hour average systolic BP; DBP24, 24 hour average diastolic BP; MAP, mean arterial pressure; SBP SD, standard deviation i.e. variability of systolic BP; DBP SD, standard deviation i.e. variability of diastolic BP; SD, standard deviation; day, daytime average BP; noct, nocturnal average BP; dip, percentage reduction from day to night BP; cIMT, carotid intima-media thickness; RHI, reactive hyperaemia index; AI@75%, EndoPAT-2000-derived augmentation index adjusted for heart rate; HR, heart rate; PWV, pulse wave velocity; PWA, pulse wave analysis; SCP systolic central pressure; DCP, diastolic central pressure.

**Table S2. Multivariable regression analyses of vascular and blood pressure related parameters.**

| **PREDICTORS** | **% FMD** | | **R^2^ adj** | | **LnRHI** | | **R^2^ adj** | | | [**AI@75 %**](about:blank) | | **R^2^ adj** | | **PWV** | | **R^2^ adj** | | **AIx  PWA** | | | **R^2^ adj** |  |  |
| --- | --- | --- | --- | --- | --- | --- | --- | --- | --- | --- | --- | --- | --- | --- | --- | --- | --- | --- | --- | --- | --- | --- | --- |
|  |  | |  | |  | |  | | |  | |  | |  | |  | |  | | |  |  |  |
| **cIMT** | -0.04 (3.57) | | NS | | -0.40 (0.32) | | NS | | | 14.3 (19.9) | | NS | | -1.38 (1.2) | | NS | | 27.7 (20.9) | | | NS |  |  |
| **% FMD** | n/a | | n/a | | 0.005 (0.007) | | NS | | | 0.33 (0.44) | | NS | | 0.02 (0.28) | | NS | | 0.054 (0.37) | | | NS |  |  |
| **LnRHI** | 0.86 (1.1) | | NS | | n/a | | n/a | | | n/a | | n/a | | -0.27 (0.37) | | NS | | -6.97 (5.0) | | | NS |  |  |
| [**AI@75 %**](about:blank) | 0.023 (0.02) | | NS | | n/a | | n/a | | | n/a | | n/a | | 0.019 (0.006) | | 0.24^#^ | | 0.34 (0.07) | | | 0.31* |  |  |
| **PWV** | 0.24 (0.27) | | NS | | -0.016 (0.02) | | NS | | | 4.11 (1.3) | | 0.24^#^ | | n/a | | n/a | | 2.88 (1.19) | | | 0.23^#^ |  |  |
| **AIx PWA** | 0.003 (0.02) | | NS | | -0.0022 (0.0016) | | NS | | | 0.39 (0.10) | | 0.30* | | 0.014 (0.006) | | 0.21^#^ | | n/a | | | n/a |  |  |
| **SCP** | 0.0018 (0.023) | | NS | | 0.0036 (0.002) | | 0.02^#^ | | | 0.65 (0.09) | | 0.43* | | 0.034 (0.007) | | 0.26* | | 0.54 (0.08) | | | 0.40* |  |  |
| **DCP** | 0.016 (0.028) | | NS | | 0.0029 (0.002) | | NS | | | 0.83 (0.11) | | 0.45* | | 0.045 (0.009) | | 0.29* | | 0.64 (0.1) | | | 0.39* |  |  |
| **SBP24** | 0.0058 (0.02) | | NS | | 0.005 (0.002) | | 0.05^#^ | | | 0.50 (0.10) | | 0.35* | | 0.027 (0.007) | | 0.22* | | 0.27 (0.09) | | | 0.27^#^ |  |  |
| **DBP24** | 0.0092 (0.031) | | NS | | 0.006 (0.002) | | 0.03^#^ | | | 0.82 (0.14) | | 0.39* | | 0.05 (0.010) | | 0.29* | | 0.55 (0.13) | | | 0.32* |  |  |
| **SBP SD** | -0.15 (0.11) | | NS | | 0.0095 (0.0089) | | NS | | | 1.54 (0.54) | | 0.22^#^ | | 0.064 (0.04) | | NS | | 1.07 (0.48) | | | 0.24^#^ |  |  |
| **DBP SD** | -0.028 (0.13) | | NS | | -0.003 (0.01) | | NS | | | 1.19 (0.60) | | 0.18^#^ | | 0.088 (0.04) | | 0.17^#^ | | 1.27 (0.53) | | | 0.25^#^ |  |  |
| **Daytime SBP** | 0.0056 (0.02) | | NS | | 0.0054 (0.0017) | | 0.06^#^ | | | 0.50 (0.10) | | 0.31* | | 0.025 (0.007) | | 0.22* | | 0.26 (0.09) | | | 0.28^#^ |  |  |
| **Daytime DBP** | 0.0067 (0.03) | | NS | | 0.0070 (0.0024) | | 0.05^#^ | | | 0.80 (0.15) | | 0.34* | | 0.047 (0.01) | | 0.27* | | 0.51 (0.13) | | | 0.31* |  |  |
| **Nighttime SBP** | 0.002 (0.023) | | NS | | 0.0047 (0.0018) | | 0.04^#^ | | | 0.37 (0.11) | | 0.23^#^ | | 0.025 (0.007) | | 0.21^#^ | | 0.23 (0.10) | | | 0.24^#^ |  |  |
| **Nighttime DBP** | 0.006 (0.03) | | NS | | 0.0038 (0.0026) | | NS | | | 0.70 (0.15) | | 0.31* | | 0.043 (0.008) | | 0.35* | | 0.46 (0.13) | | | 0.28* |  |  |
| **dip SBP %** | 0.0035 (0.058) | | NS | | 0.00027 (0.0047) | | NS | | | 0.37 (0.29) | | NS | | -0.001 (0.02) | | NS | | 0.005 (0.25) | | | NS |  |  |
| **dip DBP %** | 0.017 (0.05) | | NS | | 0.0023 (0.0036) | | NS | | | 0.063 (0.23) | | NS | | -0.011 (0.01) | | NS | | -0.072 (0.20) | | | NS |  |  |
| **HR range day** | 0.02 (0.029) | | NS | | 0.00113 (0.0015) | | NS | | | -0.007 (0.09) | | NS | | -0.005 (0.005) | | NS | | 0.92 (0.31) | | | NS |  |  |
| **HR range night** | 0.013 (0.03) | | NS | | -0.0011 (0.0026) | | NS | | | -0.28 (0.16) | | NS | | 0.0035 (0.010) | | NS | | -0.014 (0.15) | | | NS |  |  |
| **IDQ** | -0.017 (0.07) | | NS | | -0.0048 (0.0055) | | NS | | | 1.3 (0.32) | | 0.30* | | 0.041 (0.02) | | NS | | 1.01 (0.28) | | | 0.30* |  |  |
|  |  | |  | |  | |  | | |  | |  | |  | |  | |  | | |  |  |  |
|  |  | |  | |  | |  | | |  | |  | |  | |  | |  | | |  |  |  |
| **PREDICTORS** | | **SCP** | | **R^2^ adj** | | **DCP** | | **R^2^ adj** | **SBP24** | | **R^2^ adj** | | **DBP24** | | **R^2^ adj** | | **dip SBP %** | | **R^2^ adj** | **dip DBP %** | | | **R^2^ adj** |
| **cIMT** | | 8.3 (13.9) | | NS | | 1.5 (11.4) | | NS | -6.9 (15.4) | | NS | | -0.3 (10.7) | | NS | | -1.4 (6.4) | | NS | -0.17 (7.6) | | | NS |
| **% FMD** | | 0.020 (0.32) | | NS | | 0.14 (0.26) | | NS | 0.07 (0.32) | | NS | | 0.05 (0.22) | | NS | | -0.032 (0.13) | | NS | 0.06 (0.2) | | | NS |
| **LnRHI** | | 9.45 (4.5) | | 0.21^#^ | | 5.22 (3.6) | | NS | 13.9 (4.1) | | 0.18* | | 7.88 (2.95) | | 0.12^#^ | | -0.40 (1.7) | | NS | 0.77 (2.2) | | | NS |
| [**AI@75 %**](about:blank) | | 0.46 (0.06) | | 0.43* | | 0.37 (0.05) | | 0.37* | 0.33 (0.06) | | 0.26^#^ | | 0.26 (0.04) | | 0.29* | | 0.042 (0.03) | | NS | 0.0072 (0.04) | | | NS |
| **PWV** | | 5.36 (0.93) | | 0.33* | | 4.59 (0.78) | | 0.26* | 4.9 (0.98) | | 0.21^#^ | | 4.0 (0.70) | | 0.23* | | -0.03 (0.42) | | NS | -0.2 (0.5) | | | NS |
| **AIx PWA** | | 0.39 (0.068) | | 0.35* | | 0.30 (0.06) | | 0.25* | 0.22 (0.07) | | 0.16* | | 0.19 (0.05) | | 0.18* | | 0.004 (0.03) | | NS | -0.015 (0.04) | | | NS |
| **SCP** | | n/a | | n/a | | 0.679 (0.037) | | 0.73* | 0.69 (0.06) | | 0.53^#^ | | 0.50 (0.04) | | 0.55* | | 0.05 (0.03) | | NS | 0.036 (0.04) | | | NS |
| **DCP** | | 1.04 (0.06) | | 0.76* | | n/a | | n/a | 0.89 (0.07) | | 0.56^#^ | | 0.71 (0.04) | | 0.70* | | 0.016 (0.04) | | NS | -0.020 (0.05) | | | NS |
| **SBP24** | | 0.70 (0.06) | | 0.58* | | 0.585 (0.047) | | 0.56* | n/a | | n/a | | 0.62 (0.03) | | 0.80* | | 0.009 (0.03) | | NS | 0.023 (0.04) | | | NS |
| **DBP24** | | 1.03 (0.08) | | 0.60* | | 0.95 (0.05) | | 0.71* | 1.26 (0.05) | | 0.81^#^ | | n/a | | n/a | | 0.005 (0.05) | | NS | -0.016 (0.06) | | | NS |
| **SBP SD** | | 1.59 (0.41) | | 0.26* | | 1.10 (0.33) | | 0.16^#^ | 1.68 (0.39) | | 0.20^#^ | | 0.97 (0.28) | | 0.13^#^ | | 0.4 (0.16) | | 0.07^#^ | 0.37 (0.2) | | | NS |
| **DBP SD** | | 1.62 (0.46) | | 0.25^#^ | | 1.40 (0.37) | | 0.18* | 1.15 (0.45) | | 0.14* | | 0.95 (0.32) | | 0.12^#^ | | 0.12 (0.18) | | NS | 0.14 (0.2) | | | NS |
| **Daytime mean SBP** | | 0.68 (0.06) | | 0.58* | | 0.55 (0.04) | | 0.53* | n/a | | n/a | | 0.59 (0.03) | | 0.75* | | 0.056 (0.03) | | NS | 0.07 (0.04) | | | NS |
| **Daytime mean DBP** | | 1.02 (0.08) | | 0.60* | | 0.92 (0.05) | | 0.68* | 1.22 (0.06) | | 0.77^#^ | | n/a | | n/a | | 0.07 (0.05) | | NS | -1.9 (0.04) | | | 0.16* |
| **Nighttime mean SBP** | | 0.53 (0.07) | | 0.41* | | 0.49 (0.05) | | 0.43* | n/a | | n/a | | 0.53 (0.04) | | 0.63* | | -0.18 0.03) | | 0.25* | -0.18 (0.04) | | | 0.15* |
| **Nighttime mean DBP** | | 0.72 (0.10) | | 0.41* | | 0.75 (0.07) | | 0.51* | 0.95 (0.08) | | 0.54^#^ | | n/a | | n/a | | -0.23 (0.04) | | 0.21* | -0.38 (0.047) | | | 0.33* |
| **dip SBP %** | | 0.35 (0.23) | | NS | | 0.06 (0.19) | | NS | 0.047 (0.22) | | NS | | -0.005 (0.15) | | NS | | n/a | | n/a | 1.0 (0.06) | | | 0.64* |
| **dip DBP %** | | 0.18 (0.18) | | NS | | -0.051 (0.14) | | NS | 0.056 (0.18) | | NS | | -0.03 (0.12) | | NS | | 0.63 (0.04) | | 0.65* | n/a | | | n/a |
| **HR range day** | | 0.05 (0.07) | | NS | | 0.003 (0.05) | | NS | -0.059 (0.07) | | NS | | 0.003 (0.05) | | NS | | 0.029 (0.03) | | NS | 0.03 (0.03) | | | NS |
| **HR range night** | | -0.088 (0.12) | | NS | | -0.05 (0.09) | | NS | -0.069 (0.11) | | NS | | -0.065 (0.08) | | NS | | -0.11 (0.04) | | 0.06^#^ | -0.11 (0.06) | | | NS |
| **IDQ** | | 1.2 (0.24) | | 0.31* | | 1.19 (0.18) | | 0.31* | 1.29 (0.23) | | 0.23* | | 0.94 (0.16) | | 0.25* | | -0.018 (0.1) | | NS | 0.004 (0.1) | | | NS |

Confounding variables included in adjusted models included age, sex, BMI and physical activity score (IPAQ). Reported as co-efficient (standard error), and R^2^ (adjusted) value indicating goodness of fit of the model, * indicates P<0.0001, ^#^ indicates P<0.05. SBP24, 24 hour average systolic BP; DBP24, 24 hour average diastolic BP; MAP, mean arterial pressure; SBP SD, standard deviation i.e. variability of systolic BP; DBP SD, standard deviation i.e. variability of diastolic BP; SD, standard deviation; day, daytime average BP; noct, nocturnal average BP; dip, percentage reduction from day to night BP; RHI, reactive hyperaemia index; AIx, EndoPAT-2000-derived augmentation index; AI@75%, AIx adjusted for heart rate; HR, heart rate; PWV, pulse wave velocity; PWA, pulse wave analysis; SCP systolic central pressure; DCP, diastolic central pressure.

**Supplementary File 2. Early hypertension is associated with vascular stiffness rather than endothelial dysfunction in multiple regression analyses.** FMD did not show association with vascular or BP parameters, Table S2. Elevated systolic BP demonstrated augmented endothelial function, with LnRHI as a predictor of BP parameters showing positive coefficients. Measures of arterial stiffness also explained a high proportion of the variance of central and 24hr BP responses, with AI@75 demonstrating the highest R^2^ values across each of these responses, Table S2. To assess for bi-directional interactions, vascular variables were also analysed with BP as the predictor. Table S2 demonstrates that 24hr and central BP parameters accounted for a moderate proportion of the variance of AI@75, PWV and PWA AIx. Higher R^2^ values corresponded to day rather than night-time, and diastolic rather than systolic BP, in regard to both arterial stiffness measures, and central and 24hr BP parameters (Table S2). Fewer parameters accounted for the variation in percent nocturnal dip, see Table S2.

**Table S3. Comparison of Normotension (NTN), hypertension (HTN), white-coat hypertension (WCH), and masked hypertension (MHN)**.

|  | **NTN N=66 (43%)** | | | **HTN n=55 (36%)** | | **WCH N=13 (9%)** | | **MHN N=18 (12%)** | | **P value** | **R sq adj** | |
| --- | --- | --- | --- | --- | --- | --- | --- | --- | --- | --- | --- | --- |
| Male sex (%) | | 35 | (53) | 25 | (45) | 7 | (54) | 15 | (83) | 0.04 | N/A |  |
| BMI, kg/m2, mean (SD) | | 26.1 | (4.7) | 29.1 | (4.2) | 26.9 | (4.7) | 27.3 | (3.1) | 0.004 | 0.07 |  |
| Age, years, median (IQR) | | 39 | (8) | 40 | (9) | 40 | (8) | 37 | (8) | 0.6 | 0.0 |  |
| Office SBP, mmHg, Mean (SD) | | 120 | (10) | 153 | (13) | 148 | (4) | 129 | (6) | <0.001 | 0.67 |  |
| Office DBP, mmHg, mean (SD) | | 77 | (9) | 97 | (11) | 93 | (7) | 83 | (5) | <0.001 | 0.48 |  |
| Ever smoked N (%) | | 25 | (38) | 18 | (33) | 4 | (31) | 7 | (39) | 0.9 | N/A |  |
| SBP24, mmHg, mean (SD) | | 114 | (9) | 144 | (9) | 123 | (5) | 136 | (6) | <0.001 | 0.72 |  |
| DBP24, mmHg, mean (SD) | | 71 | (7) | 90 | (8) | 79 | (6) | 83 | (7.0) | <0.001 | 0.60 |  |
| SBP SD, mmHg, median (IQR) | | 9.4 | (3.4) | 117 | (3.7) | 10.4 | (2.8) | 10.5 | (3.9) | <0.001 | 0.10 |  |
| DBP SD, mmHg, median (IQR) | | 8.2 | (3.4) | 9.5 | (3.2) | 9.9 | (2.3) | 8.6 | (3.3) | 0.04 | 0.04 |  |
| % dip SBP mean (SD) | | 11.3 | (5.9) | 12.2 | (7.7) | 12.1 | (6.8) | 10.5 | (5.2) | 0.2 | 0.0 |  |
| % dip DBP mean (SD | | 15.1 | (7.3) | 16.5 | (9.7) | 16.4 | (7.6) | 14.6 | (7.8) | 0.7 | 0.0 |  |
| % FMD | | 6.0 | (4.0) | 6.7 | (4.3) | 7.0 | (4.1) | 5.9 | (2.6) | 0.7 | 0.0 |  |
| LnRHI | | 0.7 | (0.3) | 0.8 | (0.4) | 0.7 | (0.3) | 0.8 | (0.4) | 0.2 | 0.01 |  |
| Mean HR | | 62 | (19) | 68 | (24) | 68 | (21) | 64 | (26) | 0.02 | 0.05 |  |
| [AI@75 %](about:blank) | | -2.9 | (15.4) | 13.1 | (18.5) | 22.3 | (30.5) | -3.9 | (11.9) | <0.001 | 0.18 |  |
| CIMT | | 0.55 | (0.28) | 0.54 | (0.28) | 0.56 | (0.30) | 0.54 | (0.27) | 0.9 | 0.0 |  |
| PWV | | 6.4 | (2.0) | 7.4 | (2.6) | 7.7 | (3.2) | 7.2 | (0.7) | <0.001 | 0.12 |  |
| AIx % by PWA | | **1.0** | **(19.7)** | **15.3** | **(15.6)** | **18.8** | **(11.6)** | **2.1** | **(19.6)** | <0.001 | 0.13 |  |
| SCP | | 111 | (25) | 137 | (23) | 130 | (36) | 122 | (7.2) | <0.001 | 0.46 |  |
| DCP | | 76 | (18) | 95 | (17) | 88 | (26) | 86 | (5.2) | <0.001 | 0.46 |  |
| IDQ adjusted for HTN | | 6.7 | (4.5) | 8.5 | (4.6) | 9.1 | (4.9) | 7.6 | (5.3) | 0.1 | 0.02 |  |
| IPAQ | | 3437 | (2397) | 2244 | (2001) | 2858 | (1958) | 4764 | (4935) | 0.005 | 0.07 |  |

NTN, 24 hour SBP (systolic blood pressure) <130 and office SBP <140 mmHg; HTN, 24 hour SBP >130 and office SBP >140 mmHg; WCH 24hr SBP <130 and office SBP >140 mmHg; MH 24hr SBP >130 and office SBP <140 mmHg. DBP, diastolic blood pressure; CIMT, carotid IMT; LnRHI, Log reactive hyperaemia index; AIx, augmentation index; AI@75%, AIx adjusted for heart rate; SD, standard deviation; HR, heart rate; PWV, pulse wave velocity; PWA, pulse wave analysis; SCP systolic central pressure; DCP, diastolic central pressure; IDQ, interheart diet score; IPAQ, International Physical Activity Questionnaire. ANOVA or Chi^2^ testing.

**Figure S2. Correlation of brachial (24hr) and central BP by WCH and MHN subgroups:**

| 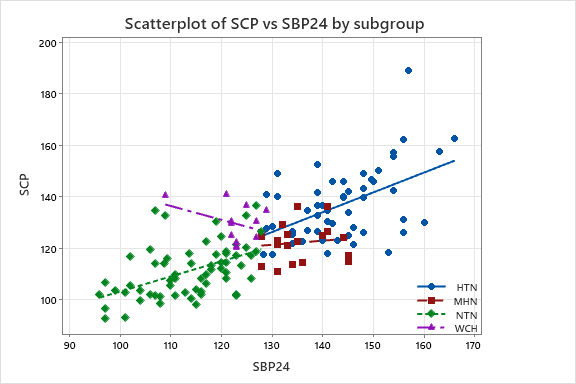 |
| --- |

SBP24, 24 hour average systolic BP; DBP24, 24 hour average diastolic BP; SCP systolic central pressure; DCP, diastolic central pressure; WCH, white coat hypertension; MHN, masked hypertension; HTN, sustained hypertension; NTN, normotension.

**Supplementary File 3. Advanced machine learning techniques**

Dimensionality reduction combined with clustering was undertaken within the hypertensive group to determine phenotypes of early hypertension based on clusters of patients in the data, with the following analytical sequence: (1) a dataset was created embedding in low-dimensional space using UMAP (explanation below); (2) Clustering reduced dimensionality space using k-means algorithm, with optimal parameters defined by Silhouette score to capture distinct groups of patients – in this case three patient groups identified as being optimal; (3) a classifier was trained on the original data using XGBoost, with class labels determined by step 2 cluster identifiers; (4) SHAP values were computed from the trained model in order to provide better interpretation of discriminating features for each of the clusters. Each methodological step is hereby described.

**U**niform **M**anifold **A**pproximation and **P**rojection (UMAP) is a dimensionality reduction technique that learns the manifold structure of data.[1] It starts by building a graph of neighbouring samples where edge weights reflect similarities (or distances) between nodes according to a given metric (e.g., Euclidean distance). Then, the embedded data is projected to lower-dimensional space while preserving its topological structure.

*K-Means* is a widely used clustering methods due to its simplicity. It partitions data into *k* groups aggregating the most similar samples. The method determines data centroids and assigns each data point to the nearest one (in terms of a given metric). The algorithm works iteratively, first initialising centroids randomly and then correcting their positions, minimising within-cluster variances.

*Silhouette Score* is a measure that allows assessing the quality of data clustering when ground-truth labels are not available.[2] Its value ranges from -1 to 1, yielding higher scores when clusters are well-separated and dense. It allows estimating the best number of clusters in data.

*Shapley values* reveal the gains and costs to measure the contribution of features to assignment to a particular class, allowing further interpretation of a decision made by a model.[3]

*Model interpretation*

To find the optimal setting, the aforementioned algorithm has been run on a grid of parameters used by the methods. The space of analysed parameters included the following: different UMAP metrics (Manhattan, Euclidean, Cosine, Correlation), the number of sample neighbours for UMAP (4, 5, 6, 7, 8, 9, 10, 15, 20), and the number of clusters for k-means (3, 4, 5). The numbers of dimensions in reduced space for UMAP was fixed to 2 and min_dist parameter of UMAP to 0 to improve clustering. The quality of clusterings for particular settings was assessed using Silhouette Score. The use of dimensionality reduction technique (UMAP) was aimed at improving separation of the clusters. The compromise between the interpretability of the model and training accuracy is maintained by using a single tree with depth up to 6. This simplistic and easy to analyse model with very low complexity obtained nearly 100% accuracy and reflected underlying structures in the data.

**Figure S3. Optimal UMAP setting: the manhattan metric, four neighbours and three k-means clusters with clustering demonstrating Silhouette score – 0.64.**


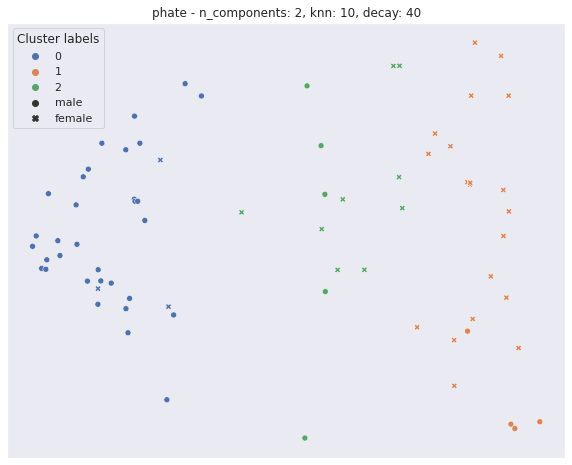


**Figure S4**. **Analysis of each cluster group by dimensional reduction using UMAP, with inclusion of SHAP values.** This demonstrates cluster characteristics of central BP as a key driver across the three clusters, augmentation index in clusters 0 and 1, 24hr DBP in clusters 0 and 1; SBP % dip and cardiovascular variation (SD, heart rate range) in clusters 0 and 2; PWV in cluster 1, and demographic features in cluster 2.

**
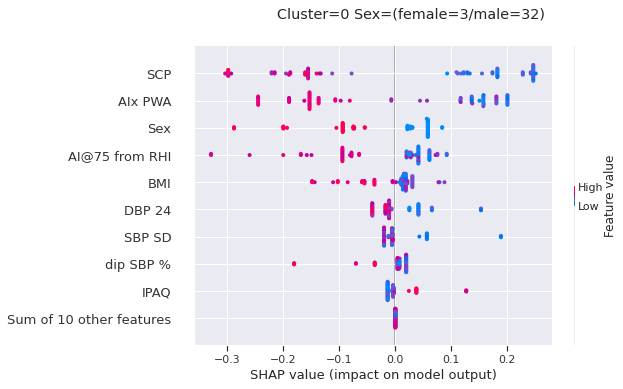
**


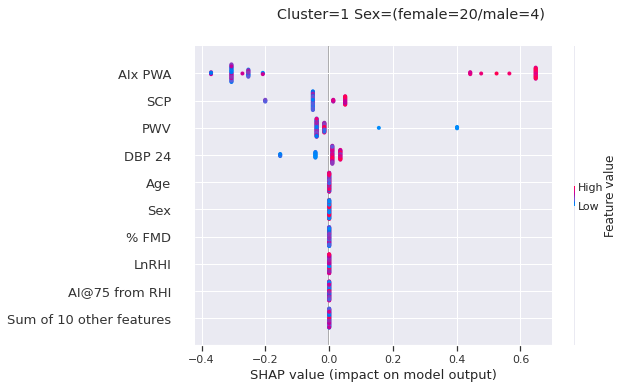


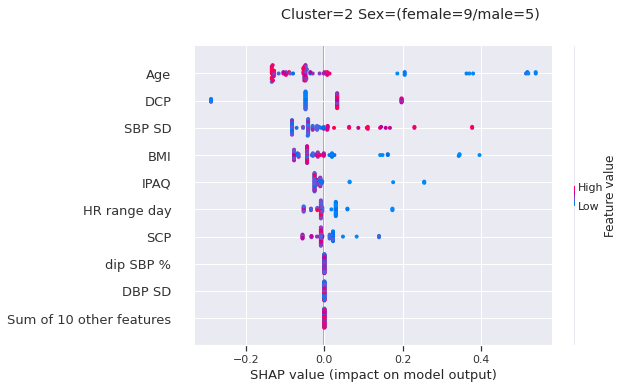


SCP, systolic central pressure; DCP, diastolic central pressure; AI@75, augmentation index adjusted to heart rate 75 derived from reactive hyperaemia index (RHI); PWV, pulse wave velocity; AIx PWA, augmentation index derived from pulse wave analysis; dip DBP %, percentage nocturnal dip in diastolic blood pressure; BMI, body mass index; HR range day, range of daytime recorded heart rates.

**References**

1 McInnes L, Healy J, Melville J. UMAP: Uniform Manifold Approximation and Projection for Dimension Reduction. Published Online First: 9 February 2018. doi:10.48550/arxiv.1802.03426

2 Rousseeuw PJ. Silhouettes: A graphical aid to the interpretation and validation of cluster analysis. *J Comput Appl Math* 1987; 20:53–65.

3 Lundberg SM, Lee S-II, Allen PG, Lee S-II. A unified approach to interpreting model predictions. In: *Advances in Neural Information Processing Systems*.; 2017. pp. 4766–4775.
